# Patient-centered outcomes for clinical trials in chronic rhinosinusitis with or without nasal polyps and allergic fungal rhinosinusitis

**DOI:** 10.1186/s41687-024-00833-6

**Published:** 2025-01-23

**Authors:** Brittany Klooster, Kaitlin Chatterton, Nazifa Ibrahim, Madison C. Bernstein, Alan L. Shields, Veleka Allen

**Affiliations:** 1 Adelphi Values, 1 Lincoln Street, Suite 2900, Boston, MA 02111 USA; 2 Sanofi US Services, Inc., Bridgewater, NJ USA

## Abstract

**Background:**

Chronic rhinosinusitis (inclusive of subtypes with nasal polyps [CRSwNP], without nasal polyps [CRSsNP], and allergic fungal rhinosinusitis [AFRS]) causes inflammation of the nose mucosa and paranasal sinuses. Unfortunately, evidence supporting use of clinical outcome assessments (COAs) in regulated clinical trials to assess key measurement concepts of these conditions is limited.

**Objective:**

To identify key disease-related symptoms and impacts, potential outcomes of interest for new treatments, and COAs available to measure those outcomes among adult and adolescent individuals living with CRSwNP, CRSsNP, and AFRS.

**Methods:**

Literature, clinical trial, and product label reviews were conducted to identify symptoms, impacts, and COAs used to assess CRSwNP, CRSsNP, and AFRS patient experiences in clinical trials. The disease related concepts identified in the literature were mapped to selected COAs to determine conceptual coverage of each COA.

**Results:**

Twenty-five articles, twenty-five clinical trial records, and four product labels were included in the review. Across conditions, nasal obstruction, nasal discharge, and altered smell were the most frequently identified symptoms. The most frequently identified impacts of CRSwNP and CRSsNP were on emotional functioning and sleep, and adopting new behaviors for AFRS. Findings for key symptoms and impacts in adolescents were limited. More than 20 COAs used in these conditions were identified, and 14 COAs (e.g., Sinonasal Outcome Test [SNOT-22]) were evaluated for conceptual coverage of the concepts identified in the literature.

**Conclusion:**

Results specify several symptom and impact outcomes, that if improved, would reflect treatment benefit for patients living with CRSwNP, CRSsNP, and/or AFRS. Several COAs demonstrated coverage of key measurement concepts and warrant further evaluation for use in clinical trials.

**Supplementary Information:**

The online version contains supplementary material available at 10.1186/s41687-024-00833-6.

## Introduction

Chronic rhinosinusitis (CRS) is a respiratory condition which causes chronic inflammation of the nose mucosa and paranasal sinuses [1] and is categorized by the presence or absence of nasal polyps (CRSwNP or CRSsNP, respectively). Within these two phenotypes, there are several subtypes including a noninvasive fungal form of CRS known as allergic fungal rhinosinusitis (AFRS; a subtype of CRSwNP) [2]. The major diagnostic criteria of both CRSsNP and CRSwNP include: (1) the presence of two or more symptoms, including nasal blockage, obstruction, or congestion, or (2) nasal discharge (anterior/posterior nasal drip), with or without facial pain/pressure, or reduction or loss of smell for ≥12 weeks [1, 3]. The major diagnostic criteria of AFRS include: (1) Type I hypersensitivity (i.e., an observed reaction to fungal antigens), (2) nasal polyposis, (3) characteristic computed tomography findings, (4) eosinophilic mucin without invasion, and (5) positive fungal stain. Other minor diagnostic criteria of AFRS include: asthma, unilateral disease, bone erosion, fungal cultures, Charcot-Leyden crystals, and serum eosinophilia [4].

The prevalence of CRSsNP ranges from 2 to 15% of the United States population, and is more common among females [5]. With CRSwNP, eosinophils infiltrate mucosal tissues and nasal polyps, and inflammatory lesions are found in the nasal airway [6, 7]. Nasal polyps are found in 1–4% of the United States general population. The typical range of diagnosis is 40–60 years, but the average onset of CRSwNP begins at 42 years [7]. AFRS accounts for about 5–10% of CRS cases and tends to affect younger people (mean patient age is 22 years) [4], although it is found in patients of all ages [2]. Clinically distinguishing between the three conditions can be done via sinus computed tomography scan or nasal endoscopy [7]. AFRS is distinguished from CRSwNP via the commonly utilized Bent and Kuhn 1994 diagnostic criteria [8].

Treatments for CRSsNP and CRSwNP include pharmaceutical and surgical interventions. Pharmaceutical interventions may include oral or topical corticosteroids, antifungals, leukotriene antagonists, omalizumab, immunotherapy, and nasal saline irrigation. Surgical intervention is typically done via endoscopic sinus surgery, which is recommended to improve symptoms such as nasal obstruction, facial pain/pressure, and post-nasal drip [1]; for patients with CRSwNP in particular, multiple operations may be required [7, 9]. Given the relative novelty of AFRS-specific diagnostic criteria, treatment generally requires both pharmaceutical and surgical intervention.

Despite the prevalence of studies investigating potential treatments for CRS conditions, evidence supporting the use of clinical outcome assessments (COAs) to assess the patient experience of symptoms and their impact on health-related quality of life in regulated clinical trials is limited in the target population [10, 11]. Whether a COA is “fit for purpose” to support medical product labeling claims depends on several factors, including evidence of the instrument’s content validity, or the extent to which the instrument measures what is relevant to a given disease or condition and that is important to patients with that disease or condition. Therefore, the primary objectives of this research are to (1) explore the primary signs, symptoms, and impacts of CRSsNP, CRSwNP, and AFRS among adolescent and adults with these conditions (i.e., individuals who may reliably self-report their experiences with a condition) to understand the broad patient experience; (2) organize the signs, symptoms, and impacts reported in the literature into conceptual models; (3) identify COAs utilized in clinical trials that measure the patient symptom and impact experience of these conditions; and (4) evaluate the extent to which identified COAs measure concepts identified in the published literature as important and relevant to the patient experience. The results presented here can help researchers understand what outcomes may be most relevant for patients with CRSsNP, CRSwNP, and AFRS and, moreover, to inform the selection or development of COAs capable of assessing these outcomes in a regulated trial environment.

## Methods

For each of the three rhinosinusitis conditions, a literature review was conducted to identify the symptoms and impacts (i.e., concepts) associated with the condition among adults (aged ≥ 18 years) and adolescents (aged 12 to <18 years). Existing COA questionnaires were identified from the articles selected as part of the literature review. A targeted search of clinical trials and product labels was also conducted to identify additional COAs that have been previously used to assess symptoms and impacts of the conditions. Concepts identified in the literature were then compared to concepts assessed by the selected COAs to determine conceptual coverage of each COA (i.e., the extent to which questionnaires measure concepts considered relevant for patients). The methodology of each of these activities is detailed in the sections below.

### Concept-focused literature review

To identify articles that reported on the signs, symptoms, and impacts associated with CRSsNP, CRSwNP, and AFRS, three separate searches were conducted in MEDLINE^®^, Embase, and PsycINFO^®^ on 19 August 2022 using the OvidSP platform. The overall search strategy was documented in detail, including keywords, limits applied, databases searched, and inclusion and exclusion criteria implemented (see full search strategies in Appendix A).

All abstracts retrieved through the search strategy were exported to Abstrackr, a web-based program [12] that allows researchers to review and tag abstracts as relevant or not relevant to the research questions. Abstracts were reviewed independently and collaboratively by three members of the research team to determine which articles should be retained or excluded for full review. It was determined a priori that up to 15 full-text articles per condition would be targeted for inclusion in the review, in consideration of prior research experience conducting concept-focused literature reviews and anticipated results in these conditions (i.e., a relatively small set of characteristic symptoms associated with numerous distal impacts). Article inclusion and exclusion decisions were based on the following criteria:

- Abstracts were **included** for full-text review if the title and/or abstracts were deemed to primarily focus on the signs, symptoms, and/or impacts associated with the condition from the perspective of adults and adolescents.
- Abstracts were **excluded** from full text review if the title and/or abstracts primarily focused on other aspects of the condition without discussion of signs, symptoms, and/or impacts; solely focused on the pathogenesis, molecular, or biology-based findings of the condition; solely discussed case studies of only a few (i.e., one or two) patients; primarily focused on non-human research; were not focused on an adult or adolescent study population; were not available in English; and/or were published prior to 2012.

The reference lists of publications selected for full-text review were also reviewed to identify additional relevant publications. Lastly, searches were also conducted in PubMed and Google Scholar to identify additional articles of interest using combinations of the search terms used in the search strategy document. Articles reporting results from qualitative studies were prioritized, though it was expected that relatively few would be available; articles reporting results from COAs completed in the target patient population (e.g., patient-reported outcome questionnaires) were also included in the review.

Once selected, information related to the study design, study sample, and CRSsNP, CRSwNP, and AFRS-related sign, symptom, and impact concepts was extracted and documented. Three separate conceptual models [13] were developed depicting the disease-related sign, symptom, and impact concepts of CRSsNP, CRSwNP, and AFRS respectively, as identified in the published literature.

### COA landscape inquiry

A COA landscape inquiry was conducted to identify COAs used in CRSsNP, CRSwNP, or AFRS clinical trials to assess patient experience of each disease. This inquiry included a review of existing clinical trials, Food and Drug Administration (FDA) labels, and articles identified in the literature review.

Clinical trials were reviewed through a targeted search conducted in ClinicalTrials.gov on 28 October 2022 using terms listed below in the “Condition or disease” field. To ensure that the search identified trials that were relevant to the study goals, the results were filtered to only include Phase 3 studies. A targeted search was also conducted across existing FDA product labels (from the DailyMed database, https://dailymed.nlm.nih.gov/dailymed/advanced-search.cfm) on 7 November 2022 via the Advanced Search Function using search terms for the condition listed below and selecting “Human Drugs” and “Indications and Usage” in the “Criteria” field.

The following search terms were used in both the review of clinical trials and FDA product labels:

- “Chronic Rhinosinusitis without Nasal Polyps”
- “CRSsNP”
- “Chronic Rhinosinusitis with Nasal Polyps”
- “CRSwNP”
- “Allergic Fungal Rhinosinusitis”
- “AFRS”
- “Fungal rhinosinusitis”

It was determined a priori that up to 15 clinical trials and up to 15 FDA product labels would be reviewed for each condition to identify the use of COAs in the evaluation of products indicated for the treatment of CRSwNP, CRSsNP, or AFRS. Product labels were excluded from review if the product was not indicated for the treatment of the condition at hand.

Certain COAs, including some commonly used to measure outcomes in these disease areas, were identified a priori as of interest and included in the concept mapping exercise: the Sinonasal Outcome Test-22 items (SNOT-22) [14], Asthma Control Questionnaire 6-item version (ACQ-6) [15], University of Pennsylvania Smell Identification Test (UPSIT) [16], Rhinosinusitis Visual Analogue Scale (VAS), and Nasal Symptom Diaries for each condition [17–19]. A short description of each COA (e.g., purpose of assessment, number of items, recall period) is provided in Appendix A. Use of these COAs by each source (i.e., literature review articles, clinical trials, and product labels) was also documented.

COAs were considered newly identified and unique if it could be confirmed based on the information provided by the trial, label, or publication that they were not the same as another identified COA. For COAs identified during the regulatory landscape inquiry to be considered for inclusion in the concept mapping exercise (as described below), COA item wording needed to be available, and the concepts measured by the COA needed to be determined relevant or of interest to the study (i.e., assessing signs, symptoms, or impacts that are important and relevant to patients with one of the specific CRS conditions). COAs excluded from further consideration were those that did not have item wording available or assessed concepts that were not of interest.

### Concept mapping exercise

Following the COA inquiry, an exercise was conducted in which the important and relevant concepts to patients with CRSwNP, CRSsNP, and/or AFRS identified in the concept-focused literature review were “mapped” (i.e., compared and contrasted) to the concepts evaluated by each of the selected COA questionnaires. This allowed researchers to determine conceptual coverage of each COA and document which and how many of the most frequently identified concepts in the literature were measured by an item of the target COA.

## Results

### CRSsNP

#### Concept-focused literature review

A total of 226 abstracts were identified via the targeted literature search. Ten of these articles were initially selected for full-text review based on the study’s inclusion and exclusion criteria, while an additional three publications were identified through the supplemental literature search. Following full-text review of these 13 publications, one was excluded due to a lack of interpretable data (i.e., the article focused on CRS but did not distinguish between CRS subtypes), resulting in 12 full-text articles being included in the literature review [5, 20–30]. Three articles included qualitative interviews with patients with CRSsNP [22, 23, 30]. A diagram outlining the flow of the literature search and review process is provided in Appendix A.

A total of 27 CRSsNP sign and symptom concepts were identified in the reviewed literature [5, 20–30]. The most frequently identified (appearing in ≥ 50.0% of articles) concepts included altered sense of smell (*n* = 12, 100.0%), followed by nasal discharge and nasal obstruction (*n* = 11 each, 91.7%), facial pain (*n* = 10, 83.3%), fatigue (*n* = 7, 58.3%), and ear pain (*n* = 6, 50.0%). Thirty-one total CRSsNP impact concepts across 11 health-related quality of life (HRQoL) domains were also identified, with the most frequently identified being feeling depressed and lack of quality sleep (*n* = 6 each, 50.0%). Overall social impact, disrupted sleep, and frustration (*n* = 5 each, 41.7%) were also frequently identified in the reviewed literature.

**Fig. 1:**
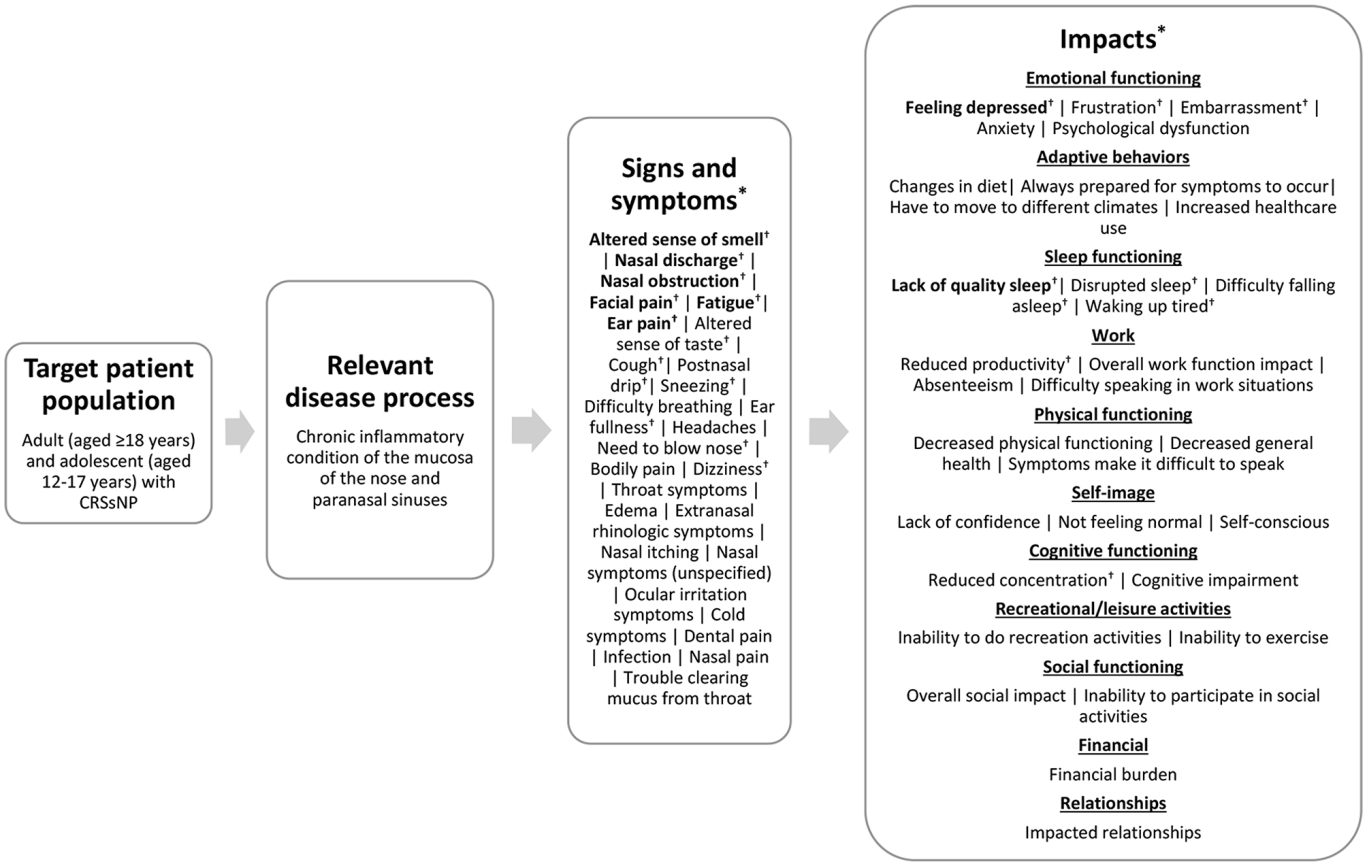
Literature-based conceptual model of chronic rhinosinusitis without nasal polyps [5, 20–30]. *Concepts shown in bolded text are the concepts most frequently identified (≥ 50.0% of articles) in the literature. ^†^Concepts were reported by Abdalla et al. [20], which included a study population of both adolescents aged 16–17 years and adult patients aged ≥ 18 years

The concepts were organized into a conceptual model presented in Fig. 1 depicting the signs, symptoms, and impacts of CRSsNP as identified in the literature. All reviewed articles (*n* = 12/12, 100%) reported data in adult populations (aged ≥ 18 years), while only one article (8.3%) reported data in adolescents aged 16–17 years (this article did not report specific results from adolescent patients). Of note, concepts are organized in the model by frequency of report.

#### COA regulatory landscape inquiry

Fourteen unique CRSsNP clinical trials were identified in the clinical trial search from which eight COAs were newly identified. One product label was identified in the FDA label search; no new COAs resulted from this step. From the concept-focused literature review, 10 COAs were identified. Further details regarding excluded trials and labels are included in Appendix A.

Of the newly identified COAs across the clinical trials, product labels, and articles in CRSsNP, seven were considered for inclusion in the concept mapping exercise based on study interest in the concepts of measurement: the Mini-Rhinoconjunctivitis Quality of Life Questionnaire (RQLQ), Rhinosinusitis Disability Index (RSDI), Patient-Reported Outcomes Measurement Information System (PROMIS) Fatigue Short Form 6a, PROMIS Sleep Disturbance Short Form 6a, PROMIS Pain Interference Short Form 6a, PROMIS Satisfaction with Participation in Social Activities Short Form, and the Chronic Rhinosinusitis Patient-Reported Outcome (CRS-PRO).

#### Concept mapping exercise

The most frequently identified symptom and impact concepts in the literature (bolded in Fig. 1 and footnoted, when covered, in Table 1) were used to understand the conceptual coverage of the 12 COAs selected for concept review.

For a summary of results from the CRSsNP concept mapping exercise, please refer to Table 1.

**Table 1:** Concept mapping of key chronic rhinosinusitis without nasal polyps symptom and impact concepts

List of COAs included in evaluation	# of symptom concepts full coverage	# of impact concepts full coverage	Total conceptual coverage	Broadest conceptual coverage
**Pre-identified COAs of interest (** ***N*** ** = 5)**				
Sinonasal Outcome Test (SNOT-22)	5/6^†,‡,§,**,††^	2/2^‡‡,§§^	7/8	✓
Rhinosinusitis Visual Analog Scale (VAS)	0/6	0/2	0/8	
CRSsNP Nasal Symptom Diary	3/6^†,‡,§^	0/2	3/8	✓ **(symptoms)**
The University of Pennsylvania Smell Identification Test (UPSIT)	1/6^*^	0/2	1/8	
Asthma Control Questionnaire (ACQ-6)	0/6	0/2	0/8	
**Additional COAs identified via clinical trial and product label review (** ***N*** ** = 7)**				
Rhinosinusitis Disability Index (RSDI) [31]	1/6^**^	2/2^‡‡,§§^	3/8	✓ **(impacts)**
PROMIS Fatigue Short Form 6a [32]	1/6^**^	0/2	1/8	
PROMIS Sleep Disturbance Short Form 6a [33, 34]	0/6	1/2^§§^	1/8	
PROMIS Pain Interference Short Form 6a [35]	0/6	0/2	0/8	
PROMIS Satisfaction with Participation in Social Activities Short Form [36]	0/6	0/2	0/8	
Chronic Rhinosinusitis Patient-Reported Outcome (CRS-PRO) [23]	5/6^*,†,‡,§,**^	0/2	7/8	✓ **(symptoms)**
Mini-Rhinoconjunctivitis Quality of Life (RQLQ) survey [37]	3/6^†,‡,**^	0/2	3/8	✓ **(symptoms)**

^*^Altered sense of smell

^†^Nasal discharge

^‡^Nasal obstruction

^§^Facial pain

^**^Fatigue

^††^Ear pain

^‡‡^Feeling depressed

^§§^Lack of quality sleep

### CRSwNP

#### Concept-focused literature review

A total of 168 abstracts were identified via the targeted literature search and subsequently reviewed against the study’s inclusion and exclusion criteria. Seven articles were selected for full-text review and an additional fifteen articles were identified through the supplemental literature search. Following a full-text review of these publications, two articles were excluded due to a lack of interpretable data (e.g., the article focused on CRS but did not distinguish between CRS subtypes). As a result, 20 full-text articles were included in the literature review [5–7, 20–30, 38–43]. Three articles included qualitative interviews with patients with CRSwNP [22, 23, 30]. A diagram outlining the flow of the literature search and review process is provided in Appendix A.

A total of 33 CRSwNP sign and symptom concepts were identified in the reviewed literature [5–7, 20–30, 38–43]. The most frequently identified concepts being altered sense of smell (*n* = 20, 100.0%), followed by nasal obstruction (*n* = 19, 95.0%), nasal discharge (*n* = 18, 90.0%), facial pain (*n* = 17, 85.0%), fatigue (*n* = 13, 65.0%), and postnasal drip (*n* = 10, 50.0%). A total of 43 CRSwNP impact concepts were identified in the literature across 13 domains. The most frequently identified impact concepts were lack of quality sleep (*n* = 10, 50.0%), followed by feeling depressed and disrupted sleep (*n* = 9 each, 45.0%), frustration (*n* = 8, 40.0%), embarrassment (*n* = 7, 35.0%), and reduced ability to concentrate and reduced productivity (*n* = 6 each, 30.0%). The most frequently identified impact domains were emotional functioning (*n* = 13, 65.0%) and sleep functioning (*n* = 13, 65.0%).

**Fig. 2:**
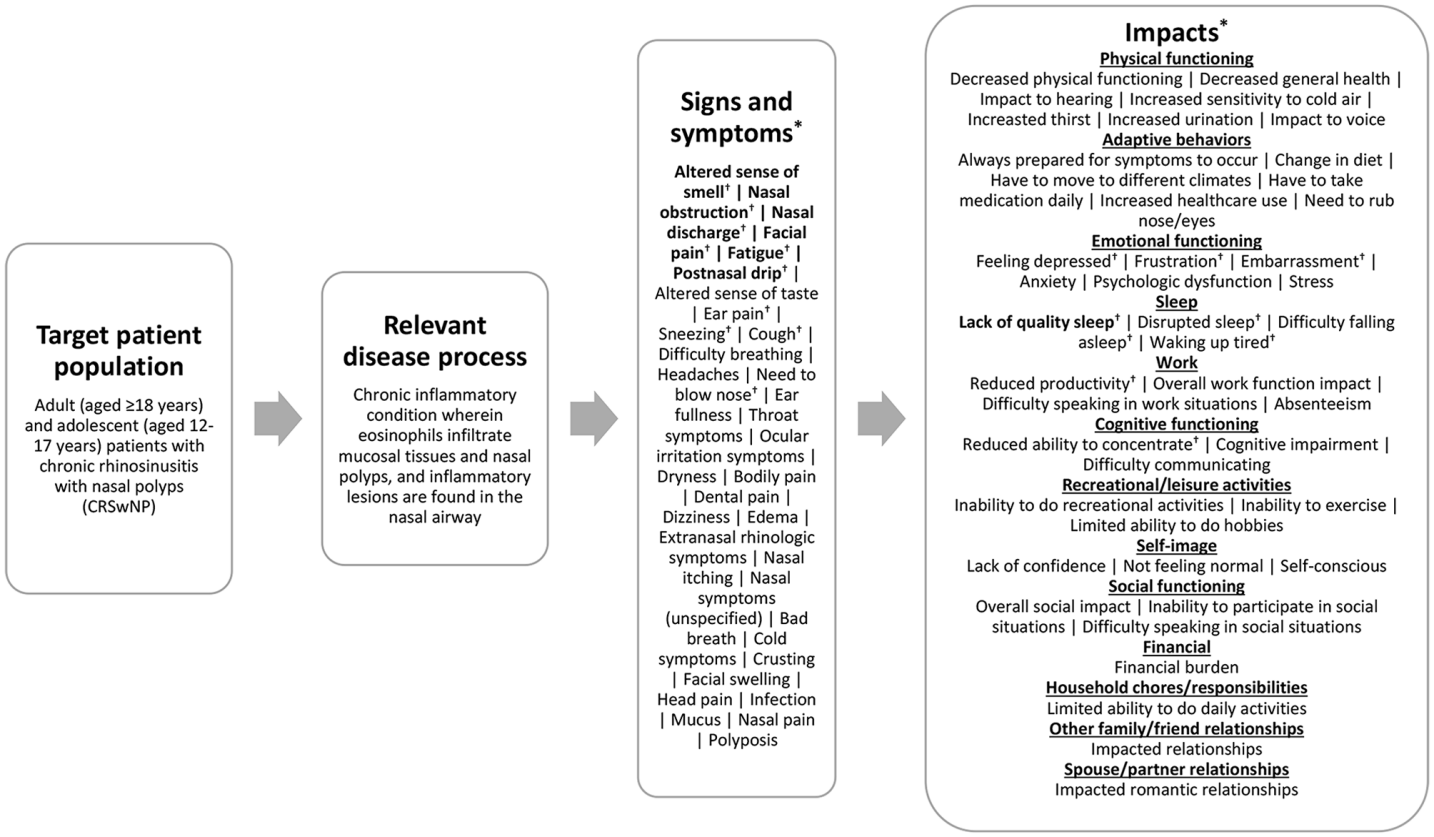
Literature-based conceptual model of chronic rhinosinusitis with nasal polyps [5–7, 20–30, 38–43]. ^*^Concepts shown in bolded text are the concepts most frequently identified (≥ 50.0% of articles) in the literature. ^†^Concepts were reported by Abdalla et al. [20], which included a study population of both adolescents aged ≥ 16 years and adult patients aged ≥ 18 years

The concepts were organized into a conceptual model presented in Fig. 2 depicting the signs, symptoms, and impacts of CRSwNP as identified in the published literature. All reviewed articles (*n* = 20/20, 100%) reported data in adult populations (aged ≥ 18 years), while only one article (5.0%) reported data in adolescents aged 16–17 years (this article did not report specific results from adolescent patients). Of note, concepts are organized in the model by frequency of report.

#### COA regulatory landscape inquiry

Seventeen unique CRSwNP clinical trials were identified in the clinical trial search from which 12 COAs were newly identified. Four unique product labels were identified via the FDA label searches from which COAs were newly identified. No COAs were identified from the literature. Further details regarding excluded trials and labels are included in Appendix A.

Of the newly identified COAs across the clinical trials and product labels, seven were considered for inclusion in the concept mapping exercise based on study interest in the concepts of measurement: Asthma Quality of Life Questionnaire (AQLQ), RSDI, PROMIS Fatigue Short Form 6a, PROMIS Sleep Disturbance Short Form 6a, PROMIS Pain Interference Short Form 6a, PROMIS Satisfaction with Participation in Social Activities Short Form 7a, and the Nasal Polyposis Symptom Diary.

#### Concept mapping exercise

The most frequently identified symptom and impact concepts in the literature (bolded in Fig. 2 and footnoted, when covered, in Table 2) were used to evaluate the conceptual coverage of the 12 COAs selected for concept review.

For key results of the CRSwNP concept mapping exercise, please refer to Table 2.

**Table 2:** Concept mapping of key chronic rhinosinusitis with nasal polyps symptom and impact concepts

List of COAs included in evaluation	# of symptom concepts full coverage	# of impact concepts full coverage	Total conceptual coverage	Broadest conceptual coverage
**Pre-identified COAs of interest (** ***N*** ** = 5)**				
Sinonasal Outcome Test (SNOT-22)	5/6^†,‡,§,**,††^	1/1^‡‡^	6/7	✓
Rhinosinusitis Visual Analog Scale (VAS)	0/6	0/1	0/7	
CRSwNP Nasal Symptom Diary	3/6^†,‡,§^	0/1	3/7	✓ **(symptoms)**
The University of Pennsylvania Smell Identification Test (UPSIT)	1/6^*^	0/1	1/7	
Asthma Control Questionnaire (ACQ-6)	0/6	0/1	0/7	
**Additional COAs identified via clinical trial and product label review (** ***N*** ** = 7)**				
Asthma Quality of Life Questionnaire (AQLQ)	0/6	0/1	0/7	
Rhinosinusitis Disability Index (RSDI)	1/6^**^	1/1^‡‡^	2/7	✓ **(impacts)**
PROMIS Fatigue Short Form 6a	1/6^**^	0/1	1/7	
PROMIS Sleep Disturbance Short Form 6a	0/6	1/1^‡‡^	1/7	✓ **(impacts)**
PROMIS Pain Interference Short Form 6a	0/6	0/1	0/7	
PROMIS Satisfaction with Participation in Social Activities Short Form	0/6	0/1	0/7	
Nasal Polyposis Symptom Diary	5/6^*,†,‡,§,††^	0/1	5/7	✓ **(symptoms)**

^*^Altered sense of smell

^†^Nasal obstruction

^‡^Nasal discharge

^§^Facial pain

^**^Fatigue

^††^Postnasal drip

^‡‡^Lack of quality sleep

### AFRS

#### Concept-focused literature review

A total of 209 potentially relevant abstracts were identified via the targeted literature search and subsequently reviewed against the study’s inclusion and exclusion criteria. Seven articles were selected for full-text review and six additional publications were identified through the supplemental literature search. Following a full-text review of these 13 publications, seven were excluded due to lack of interpretable data (e.g., the article did not distinguish between AFRS and other allergic/eosinophilic inflammatory sinus conditions). As a result, six full-text articles were included in the literature review [9, 22, 44–47]. One article included qualitative interviews with patients with AFRS [22]. A diagram outlining the flow of the literature search and review process is provided in Appendix A.

A total of 18 AFRS sign and symptom concepts were identified in the reviewed literature [9, 22, 44–47]. The most frequently identified (appearing in ≥ 50.0% of articles) concepts included nasal discharge (*n* = 6, 100.0%), followed by nasal obstruction (*n* = 5, 83.3%), headache (*n* = 4, 66.7%), altered sense of smell and post-nasal drip (each *n* = 3, 50.0%). A total of seven AFRS impact concepts (across six HRQoL domains) were identified in the literature. Impact concepts included changes in diet, having to move to different climates, financial burden, impacted relationships, disrupted sleep, overall social impact, and overall work impact (*n* = 1 each, 16.7%).

**Fig. 3:**
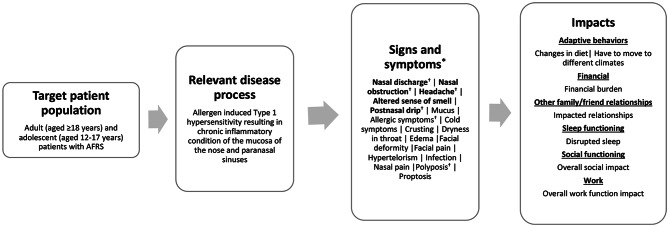
Literature-based conceptual model of allergic fungal rhinosinusitis [9, 22, 44–47]. *Abbreviation AFRS* allergic fungal rhinosinusitis. *Concepts shown in bolded text are the concepts most frequently identified (≥ 50.0% of articles) in the literature. ^†^Concepts were reported by Rojita et al. [45] and Verma et al. [47] which included a study population of adult patients aged ≥ 18 years and adolescents aged 11–17 years (Rojita et al.) and children and adolescents aged 7–17 years (Verma et al.)

The concepts were organized into a conceptual model presented in Fig. 3 depicting the signs, symptoms, and impacts of AFRS as identified in the published literature. Of the reviewed articles, three articles (50.0%) did not specify whether the literature reviewed included adults and/or adolescents. Two articles (33.3%) reported data in adult, adolescent, and/or child populations (aged 11–50 years and aged 7–65 years) but did not report specific results for each population. One article reported data only in an adult (aged ≥ 18 years) population. Of note, concepts are organized in the model by frequency of report.

#### COA regulatory landscape inquiry

Four unique AFRS clinical trials were identified in the clinical trial search, from which four COAs were newly identified. No product labels and therefore no new COAs were identified in the FDA label search. No COAs were newly identified from the literature. Of the four newly identified COAs, the RSDI was selected for inclusion in the concept mapping exercise based on study interest in the concepts of measurement.

#### Concept mapping exercise

The most frequently identified symptom concepts as well as each impact concept in the literature (bolded in Fig. 3 and footnoted, when covered, in Table 3) were used to evaluate the conceptual coverage of the six COAs selected for concept review.

For key results of the AFRS concept mapping exercise, please refer to Table 3.

**Table 3:** Concept mapping of key allergic fungal rhinosinusitis symptom and impact concepts

List of COAs included in evaluation	# of symptom concepts full coverage	# of impact concepts full coverage	Total conceptual coverage	Broadest conceptual coverage
**Pre-identified COAs of interest (** ***N*** ** = 5)**				
Sinonasal Outcome Test (SNOT-22)	3/5^*,†,§^	2/7^**,§§^	5/12	✓
Rhinosinusitis Visual Analog Scale (VAS)	0/5	0/7	0/12	
AFRS Nasal Symptom Diary	3/5^*,†,‡^	0/7	3/12	✓ **(symptoms)**
The University of Pennsylvania Smell Identification Test (UPSIT)	1/5^‡^	0/7	1/12	
Asthma Control Questionnaire (ACQ-6)	0/5	0/7	0/12	
**Additional COAs identified via clinical trial review (** ***N*** ** = 1)**				
Rhinosinusitis Disability Index (RSDI)	0/5	2/7^††,‡‡^	2/12	

^*^Nasal discharge

^†^Nasal obstruction

^‡^Altered sense of smell

^§^Postnasal drip

^**^Disrupted sleep

^††^Impacted relationships

^‡‡^Overall social impact

^§§^Overall work function impact

## Discussion

Results from this concept-focused literature review inform a deeper understanding of the unique patient experience of CRSsNP, CRSwNP, and AFRS from the perspective of the literature. Additionally, three novel, distinct conceptual models outlining the signs, symptoms, and impacts of each condition were developed based on the literature review results, which were not previously found in the literature at the time this research was conducted. These models provide insight into concepts of measurements for inclusion in measurement strategies for clinical trials and can be interpreted as the disease-related experiences that patients may most wish to see improve.

The literature review findings demonstrate that several overlapping symptom and impact concepts may be considered for inclusion in an overall CRS measurement strategy, but also that some symptom and impact concepts are unique to CRSsNP, CRSwNP, and AFRS and should be considered in disease-specific measurement strategies. Overlapping symptom concepts identified most frequently across the three conditions included: altered sense of smell, nasal discharge, nasal obstruction, facial pain, postnasal drip, and headaches. Of these, altered sense of smell, nasal discharge, and nasal obstruction were most frequently identified for all three conditions.

While identified for all conditions, postnasal drip was only frequently identified across CRSwNP and AFRS, and headaches were only frequently identified for AFRS. Facial pain and fatigue were only identified across CRSsNP and CRSwNP and were among the most frequently identified symptom concepts for both conditions. Likewise, ear pain was only identified across CRSsNP and CRSwNP but was only frequently identified for CRSsNP.

Overlapping HRQoL impact concepts frequently identified across the three conditions included: changes in diet, having to move to different climates, disrupted sleep, overall work function impact, financial burden, overall social function impact, and impacted relationships. All impact concepts identified for AFRS were identified in one article from the literature review and overlapped with CRSsNP and CRSwNP concepts, therefore it is difficult to draw conclusions regarding the unique impacts to patients’ lives associated with AFRS symptoms. The most frequently identified impact concept across CRSsNP and CRSwNP was lack of quality sleep. The impact concept of feeling depressed was also frequently identified for CRSsNP.

Moreover, all impact concepts identified in the literature for CRSsNP overlapped with CRSwNP. Other impact concepts identified for CRSwNP alone included stress, have to take medication daily, need to rub nose/eyes, impact to hearing, increased sensitivity to cold air, increased thirst, increased urination, impact to voice, limited ability to do hobbies, difficulty speaking in social situations, limited ability to do daily activities, and impacted romantic relationships (though none were identified in ≥50.0% of the articles). Additionally, more impact concepts related to the domains of physical functioning and adaptive behaviors were identified in the literature for CRSwNP compared to CRSsNP.

The previously identified COAs were the same across all three conditions, except for the condition-specific nasal symptom diaries. Among these COAs, the SNOT-22 had the broadest conceptual coverage across the key symptoms and impacts for each condition and each condition-specific nasal symptom diary demonstrated coverage of several of the key symptoms.

Among the newly identified COAs, the CRS-PRO (*n* = 5/6) and RQLQ (*n* = 3/6) demonstrated coverage of key symptoms, and the RSDI (*n* = 2/2) and PROMIS Sleep Disturbance Short Form 6a (*n* = 1/2) broadly covered key impact concepts for CRSsNP.

Similarly, for CRSwNP, the Nasal Polyposis Symptom Diary (*n* = 5/6) demonstrated coverage of key symptoms; key impact concepts were broadly covered by the RSDI (*n* = 1/1) and PROMIS Sleep Disturbance Short Form 6a (*n* = 1/1). The RSDI was the only newly identified COA used in an AFRS patient population and covered some AFRS impact concepts (*n* = 2/6).

For CRSsNP, altered sense of smell, nasal discharge, nasal obstruction, feeling depressed, and lack of quality sleep emerge as important concepts which may be considered for inclusion in a measurement strategy. Because they contain items broadly assessing these concepts, the CRSsNP Nasal Symptom Diary, CRS-PRO, SNOT-22, and RSDI may be considered for inclusion in a CRSsNP COA measurement strategy.

For CRSwNP, altered sense of smell, nasal obstruction, nasal discharge, and lack of quality of sleep emerge as important concepts which may be considered for inclusion in a measurement strategy. Because they contain items broadly assessing these concepts, the Nasal Polyposis Symptom Diary, SNOT-22, RSDI, and CRSwNP Nasal Symptom Diary may be considered for inclusion in a CRSwNP COA measurement strategy.

For AFRS, nasal discharge and nasal obstruction emerge as important concepts which may be considered for inclusion in a measurement strategy. Because they contain items broadly assessing these concepts, the AFRS Nasal Symptom Diary, SNOT-22, and RSDI may be considered for inclusion in a AFRS COA measurement strategy.

COA measurement strategies for CRSsNP, CRSwNP, and AFRS found in the literature share similarities with the findings of this work. The SNOT-22 and SNOT-20 (a variation of the SNOT-22) have been administered in clinical studies evaluating corticosteroids, which is one of the main non-surgical treatments for CRSsNP, CRSwNP, and AFRS [48, 49]. Studies have also utilized a daily symptom diary individual scores for rhinorrhea, postnasal drip, nasal obstruction, facial pain or pressure, and headache and a total score to evaluate treatment efficacy in CRSsNP and CRSwNP [50]. Omalizumab, an antibiotic indicated for treatment of both AFRS and CRSwNP [9, 51], utilized the AQLQ and ACQ as part of its COA measurement strategy [52]. Additionally, treatment for AFRS includes antifungals such as itraconazole and is frequently assessed using the SNOT-20 as part of AFRS measurement strategies. Treatments for CRS are also indicated for chronic sinusitis and allergic rhinitis, and those clinical programs have included the SNOT-20, RQLQ, and RSDI in their COA measurement strategies [53, 54].

Limitations of this work include that most of the reviewed articles in the literature review presented data derived from questionnaires that were administered to individuals with each condition, rather than data collected from patients through qualitative interviews. The quality of qualitative studies included in this review was not evaluated. The concepts reported from this research are from the literature published between 2012 and 2022. Another limitation is the lack of literature findings for the adolescent patient experience (aged 12 − 17 years). Articles that reported results in adolescent patients did not indicate whether concepts were specific to the adolescent experience. Additionally, this review identified few AFRS-specific articles in the literature and therefore the literature-based understanding of the AFRS patient experience is limited. Articles more focused on CRS overall often overlapped between the key symptom and impact concepts of CRSsNP and CRSwNP and did not clarify the two diseases in literature and reported concepts, limiting the understanding of each unique disease experience. Lastly, the COA landscape inquiry was limited to a targeted review of the existing literature, clinical trials, or product labels to identify the use of COAs within these CRS conditions; additional COAs that measure concepts of interest may exist. Recommendations for future research include conducting qualitative interviews with each patient population to identify the most important and relevant concepts to patients with each condition, including capturing the adolescent experience and an evaluation of the psychometric properties and interpretation of scores of COAs used in these conditions.

## Conclusions

Given the impact CRS-related symptoms exert on patients, results from this research specify several symptom and impact outcomes that, if improved, would reflect benefit for adult patients living with these conditions. Individuals with CRSsNP, CRSwNP, or AFRS experience similar, though not always the same, symptoms and impacts due to their conditions; altered sense of smell, nasal discharge, nasal obstruction, disrupted sleep, and overall social impact emerged from this research as core symptoms and impacts which should be considered for measurement in all three conditions. The experience of adolescent patients was not well documented in the reviewed literature; thus, further research is needed in this area. Findings from these research activities can be used to inform the development of COA measurement strategies for clinical programs developing treatments for these diseases. While the SNOT-22 demonstrated the broadest conceptual coverage of the key symptoms and impacts across each condition, several COAs emerged as potentially appropriate for use in CRSsNP/CRSwNP/AFRS trials to support clinical endpoints. Results suggest that multiple assessments may more comprehensively measure the experiences that are important to patients with these three sinonasal conditions.

## Electronic supplementary material

Below is the link to the electronic supplementary material.

Supplementary Material 1

## Data Availability

All data generated or analysed during this study are included in this published article and its supplementary information files.

## Abbreviations

ACQ: Asthma Control Questionnaire
AFRS: Allergic fungal rhinosinusitis
AQLQ: Asthma Quality of Life Questionnaire
COA: Clinical outcome assessment
CRS-PRO: Chronic Rhinosinusitis Patient-Reported Outcome
CRSsNP: Chronic rhinosinusitis without nasal polyps
CRSwNP: Chronic rhinosinusitis with nasal polyps
FDA: Food and Drug Administration
HRQoL: Health-related quality of life
PROMIS: Patient-Reported Outcomes Measurement Information System
RQLQ: Rhinoconjunctivitis Quality of Life Questionnaire
RSDI: Rhinosinusitis Disability Index
SNOT: Sinonasal Outcome Test
UPSIT: University of Pennsylvania Smell Identification Test
VAS: Visual Analog Scale

## References

[1] Slovick A, Long J, Hopkins C (2014) Updates in the management of chronic rhinosinusitis. Future Med 6:649–663

[2] Hoyt AEW, Borish L, Gurrola J, Payne S (2016) Allergic fungal rhinosinusitis. J Allergy Clin Immunology: Pract 4(4):599–604. 10.1016/j.jaip.2016.03.010 27393774 10.1016/j.jaip.2016.03.010

[3] Fokkens WJ, Lund VJ, Hopkins C et al (2020) European position paper on Rhinosinusitis and nasal polyps. Rhinology 58(29):1–464 32077450 10.4193/Rhin20.600

[4] Glass D, Amedee RG (2011) Allergic fungal rhinosinusitis: a review. Ochsner J Fall 11(3):271–275 PMC3179194 21960761

[5] Talat R, Speth MM, Gengler I et al (2020) Chronic Rhinosinusitis patients with and without polyps experience different Symptom Perception and Quality of Life burdens. Am J Rhinol Allergy Nov 34(6):742–750. 10.1177/1945892420927244 10.1177/1945892420927244 32437223

[6] Gelardi M, Bocciolini C, Notargiacomo M et al (2022) Chronic rhinosinusitis with nasal polyps: how to identify eligible patients for biologics in clinical practice. Acta Otorhinolaryngol Ital Feb 42(1):75–81. 10.14639/0392-100x-n1699 10.14639/0392-100X-N1699 PMC9058935 35292789

[7] Stevens WW, Schleimer RP, Kern RC (2016) Chronic rhinosinusitis with Nasal Polyps. The journal of allergy and clinical immunology in practice. Jul-Aug 4(4):565–572. 10.1016/j.jaip.2016.04.012 10.1016/j.jaip.2016.04.012 PMC4939220 27393770

[8] Bent JP 3rd, Kuhn FA (1994) Diagnosis of allergic fungal sinusitis. Otolaryngol Head Neck Surg Nov 111(5):580–588. 10.1177/019459989411100508 10.1177/019459989411100508 7970796

[9] Dykewicz MS, Rodrigues JM, Slavin RG (2018) Allergic fungal rhinosinusitis. J Allergy Clin Immunol 142(2):341–351. 10.1016/j.jaci.2018.06.023 10.1016/j.jaci.2018.06.023 30080526

[10] Rudmik L, Hopkins C, Peters A, Smith TL, Schlosser RJ, Soler ZM (2015) Patient-reported outcome measures for adult chronic rhinosinusitis: a systematic review and quality assessment. J Allergy Clin Immunol 136(6):1532–1540 e2 26654198 10.1016/j.jaci.2015.10.012

[11] Mercieca-Bebber R, King MT, Calvert MJ, Stockler MR, Friedlander M (2018) The importance of patient-reported outcomes in clinical trials and strategies for future optimization. Patient Relat Outcome Measures 9:353–367. 10.2147/prom.s156279 10.2147/PROM.S156279 PMC6219423 30464666

[12] Agency for Healthcare Research and Quality (2012) Abstrackr: software for semi-automatic citation screening. Accessed 28 June 2024. https://effectivehealthcare.ahrq.gov/topics/abstractr/abstract

[13] Wilson IB, Cleary PD (1995) Linking clinical variables with health-related quality of life. A conceptual model of patient outcomes. JAMA 273(1):59–65 7996652

[14] Washington University (2006) Sino-nasal outcome test (SNOT-22). Accessed 4 May 2023. https://www.canvasc.ca/wp-content/uploads/2021/10/SNOT22.pdf

[15] Juniper EF, O’Byrne PM, Guyatt GH, Ferrie PJ, King DR (1999) Development and validation of a questionnaire to measure asthma control. Eur Respir J 10/1999 14(4):902–907 10.1034/j.1399-3003.1999.14d29.x 10573240

[16] University of Pennsylvania (2023) University of Pennsylvania Smell Identification Test (UPSIT). Accessed 4 May 2023. https://www.reginfo.gov/public/do/DownloadDocument?objectID=25141601

[17] ClinicalTrials.gov (2024) Dupilumab in CRSsNP (Liberty CRSsNP). Accessed 23 Feb 2024. https://clinicaltrials.gov/study/NCT04678856?cond=CRSsNP%26spons=Sanofi%26rank=1

[18] ClinicalTrials.gov (2019) Controlled clinical study of Dupilumab in patients with nasal polyps (SINUS-52). Accessed 23 Feb 2024. https://clinicaltrials.gov/study/NCT02898454?cond=crswnp%26spons=Sanofi%26rank=2

[19] ClinicalTrials.gov (2024) Dupilumab in allergic fungal rhinosinusitis (AFRS) (LIBERTY-AFRS-AI). Accessed 23 Feb 2024. https://clinicaltrials.gov/study/NCT04684524?cond=aFRS%26spons=Sanofi%26rank=1

[20] Abdalla S, Alreefy H, Hopkins C (2012) Prevalence of sinonasal outcome test (SNOT-22) symptoms in patients undergoing surgery for chronic rhinosinusitis in the England and Wales National prospective audit. Clin Otolaryngol Aug 37(4):276–282. 10.1111/j.1749-4486.2012.02527.x 10.1111/j.1749-4486.2012.02527.x 22776038

[21] Alt JA, Mace JC, Smith TL, Soler ZM (2016) Endoscopic sinus surgery improves cognitive dysfunction in patients with chronic rhinosinusitis. Int Forum Allergy Rhinol Dec 6(12):1264–1272. 10.1002/alr.21820 10.1002/alr.21820 PMC5140732 27384037

[22] Erskine SE, Notley C, Wilson AM, Philpott CM (2014) Managing chronic rhinosinusitis and respiratory disease: a qualitative study of triggers and interactions. J Asthma: Official J Association Care Asthma 52(6):600–605. 10.3109/02770903.2014.995308 10.3109/02770903.2014.995308 25539398

[23] Ghadersohi S, Price CPE, Jensen SE et al (2020) Development and preliminary validation of a new patient-reported outcome measure for chronic Rhinosinusitis (CRS-PRO). The journal of allergy and clinical immunology in practice. Jul-Aug 8(7):2341–2350e1. 10.1016/j.jaip.2020.04.048 10.1016/j.jaip.2020.04.048 PMC7448958 32376490

[24] Gregurić T, Trkulja V, Baudoin T, Grgić M, Šmigovec I, Kalogjera L (2016) Differences in the sino-nasal outcome test 22 and visual analog scale symptom scores in chronic rhinosinusitis with and without nasal polyps. Am J Rhinol Allergy Mar-Apr 30(2):107–112. 10.2500/ajra.2016.30.4274 10.2500/ajra.2016.30.4274 26980391

[25] Hanif T, Laulajainen-Hongisto A, Luukkainen A et al (2020) Hierarchical clustering in evaluating inflammatory upper airway phenotypes; increased symptoms in adults with allergic multimorbidity. Asian Pac J Allergy Immunol Dec 38(4):239–250. 10.12932/ap-170818-0395 10.12932/AP-170818-0395 31175712

[26] McCann AC, Trope M, Walker VL et al (2021) Olfactory dysfunction is not a determinant of patient-reported chronic Rhinosinusitis Disease Control. Laryngoscope Jul 131(7):E2116–e2120. 10.1002/lary.29280 10.1002/lary.29280 33300623

[27] Nilsen AH, Helvik AS, Thorstensen WM, Salvesen Ø, Bugten V (2019) General health, vitality, and social function after sinus surgery in chronic rhinosinusitis. Laryngoscope Investig Otolaryngol Oct 4(5):476–483. 10.1002/lio2.299 10.1002/lio2.299 PMC6793610 31637289

[28] Palmer JN, Messina JC, Biletch R, Grosel K, Mahmoud RA (2019) A cross-sectional, population-based survey of U.S. adults with symptoms of chronic rhinosinusitis. Allergy Asthma Proc. 40(1):48–56. 10.2500/aap.2019.40.4182 10.2500/aap.2019.40.4182 30582496

[29] Thompson CF, Price CP, Huang JH et al (2016) A pilot study of symptom profiles from a polyp vs an eosinophilic-based classification of chronic rhinosinusitis. Int Forum Allergy Rhinol May 6(5):500–507. 10.1002/alr.21687 10.1002/alr.21687 PMC4856570 26683389

[30] Vennik J, Eyles C, Thomas M et al (2019) Chronic rhinosinusitis: a qualitative study of patient views and experiences of current management in primary and secondary care. BMJ Open Apr 23(4):e022644. 10.1136/bmjopen-2018-022644 10.1136/bmjopen-2018-022644 PMC6501991 31015263

[31] Benninger MS, Senior BA (1997) The development of the Rhinosinusitis Disability Index. Arch Otolaryngol Head NeckSurg 11/1997 123(11):1175–1179 10.1001/archotol.1997.01900110025004 9366696

[32] Lai JS, Cella D, Choi S et al (2011) How item banks and their application can influence measurement practice in rehabilitation medicine: a PROMIS fatigue item bank example. Arch Phys Med Rehabil Oct 92(10 Suppl):S20–S27. 10.1016/j.apmr.2010.08.033 10.1016/j.apmr.2010.08.033 PMC3696589 21958919

[33] Buysse DJ, Yu L, Moul DE et al (2010) Development and validation of patient-reported outcome measures for sleep disturbance and sleep-related impairments. Sleep Jun 33(6):781–792. 10.1093/sleep/33.6.781 10.1093/sleep/33.6.781 PMC2880437 20550019

[34] Yu L, Buysse DJ, Germain A et al (2011) Development of short forms from the PROMIS™ sleep disturbance and sleep-related impairment item banks. Behav Sleep Med Dec 28(1):6–24. 10.1080/15402002.2012.636266 10.1080/15402002.2012.636266 PMC3261577 22250775

[35] Amtmann D, Cook KF, Jensen MP et al (2010) Development of a PROMIS item bank to measure pain interference. Pain Jul 150(1):173–182. 10.1016/j.pain.2010.04.025 10.1016/j.pain.2010.04.025 PMC2916053 20554116

[36] Hahn EA, Devellis RF, Bode RK et al (2010) Measuring social health in the patient-reported outcomes measurement information system (PROMIS): item bank development and testing. Qual Life Res 19(7):1035–1044. 10.1007/s11136-010-9654-0 10.1007/s11136-010-9654-0 PMC3138729 20419503

[37] Juniper EF, Thompson AK, Ferrie PJ, Roberts JN (2000) Development and validation of the mini Rhinoconjunctivitis Quality of Life Questionnaire. Clin Exp Allergy 1/2000 30(1):132–140 10.1046/j.1365-2222.2000.00668.x 10606940

[38] Campion NJ, Kohler R, Ristl R, Villazala-Merino S, Eckl-Dorna J, Niederberger-Leppin V (2021) Prevalence and Symptom Burden of Nasal Polyps in a large Austrian Population. The journal of allergy and clinical immunology in practice. Nov 9(11):4117–4129e2. 10.1016/j.jaip.2021.06.037 10.1016/j.jaip.2021.06.037 34265447

[39] Khan AH, Abbe A, Falissard B et al (2021) Data Mining of Free-text responses: an innovative Approach to analyzing patient perspectives on treatment for chronic rhinosinusitis with Nasal Polyps in a phase IIa proof-of-Concept Study for Dupilumab. Patient Prefer Adherence 15:2577–2586. 10.2147/ppa.S320242 34848949 10.2147/PPA.S320242 PMC8611726

[40] Lee SE, Hopkins C, Mullol J et al (2022) Dupilumab improves health related quality of life: results from the phase 3 SINUS studies. Allergy Jul 77(7):2211–2221. 10.1111/all.15222 10.1111/all.15222 PMC9305228 35034364

[41] O’Quinn S, Shih VH, Martin UJ et al (2022) Measuring the patient experience of chronic rhinosinusitis with nasal polyposis: qualitative development of a novel symptom diary. Int Forum Allergy Rhinol Aug 12(8):996–1005. 10.1002/alr.22952 10.1002/alr.22952 PMC9543166 34921526

[42] Peters AT, Han JK, Hellings P et al (2021) Indirect treatment comparison of biologics in chronic rhinosinusitis with nasal polyps. J Allergy Clin Immunol Pract 9(6):2461–2471.e5. 10.1016/j.jaip.2021.01.031 10.1016/j.jaip.2021.01.031 33548517

[43] Xu Z, Luo X, Xu L et al (2020) Effect of short-course glucocorticoid application on patients with chronic rhinosinusitis with nasal polyps. World Allergy Organ J Jun 13(6):100131. 10.1016/j.waojou.2020.100131 10.1016/j.waojou.2020.100131 PMC7300158 32566071

[44] AlAhmari AA (2021) Allergic fungal rhinosinusitis in Saudi Arabia: a review of recent literature. Cureus Dec 13(12):e20683. 10.7759/cureus.20683 10.7759/cureus.20683 PMC8785804 35106223

[45] Rojita M, Samal S, Pradhan P, Venkatachalam VP (2017) Comparison of Steroid and Itraconazole for Prevention of recurrence in allergic fungal rhinosinusitis: a Randomized Controlled Trial. J Clin Diagn Res Apr 11(4):Mc01–mc03. 10.7860/jcdr/2017/23488.9610 10.7860/JCDR/2017/23488.9610 PMC5449820 28571174

[46] Singh V (2019) Fungal rhinosinusitis: unravelling the Disease Spectrum. J Maxillofac Oral Surg Jun 18(2):164–179. 10.1007/s12663-018-01182-w 10.1007/s12663-018-01182-w PMC6441414 30996535

[47] Verma RK, Patro SK, Francis AA, Panda NK, Chakrabarti A, Singh P (2017) Role of preoperative versus postoperative itraconazole in allergic fungal rhinosinusitis. Med Mycol Aug 1(6):614–623. 10.1093/mmy/myw125 10.1093/mmy/myw125 27838640

[48] Yamasaki A, Hoehle LP, Phillips KM et al (2018) Association between systemic antibiotic and corticosteroid use for chronic rhinosinusitis and quality of life. Laryngoscope Jan 128(1):37–42. 10.1002/lary.26778 10.1002/lary.26778 28731529

[49] Salil A, Joy N, Faizal B (2023) A prospective study comparing itraconazole alone versus systemic steroids alone as adjuncts to topical steroids in the post-operative management of allergic fungal rhinosinusitis. Clin Otolaryngol Mar 48(2):356–362. 10.1111/coa.14014 10.1111/coa.14014 36478077

[50] Chong LY, Head K, Hopkins C, Philpott C, Schilder AG, Burton MJ (2016) Intranasal steroids versus placebo or no intervention for chronic rhinosinusitis. Cochrane Database Syst Rev Apr 26(4):Cd011996. 10.1002/14651858.CD011996.pub2 10.1002/14651858.CD011996.pub2 PMC9393647 27115217

[51] Omalizumab [package insert] (2023) https://www.accessdata.fda.gov/drugsatfda_docs/label/2023/103976Orig1s5243lbl.pdf

[52] Henriksen DP, Bodtger U, Sidenius K et al (2020) Efficacy of omalizumab in children, adolescents, and adults with severe allergic asthma: a systematic review, meta-analysis, and call for new trials using current guidelines for assessment of severe asthma. Allergy Asthma Clin Immunol 16:49. 10.1186/s13223-020-00442-0 32565844 10.1186/s13223-020-00442-0 PMC7302157

[53] Meltzer EO (2001) Quality of life in adults and children with allergic rhinitis. J Allergy Clin Immunol Jul 108(1 Suppl):S45–53. 10.1067/mai.2001.115566 10.1067/mai.2001.115566 11449206

[54] Linder JA, Singer DE, Ancker M, Atlas SJ (2003) Measures of health-related quality of life for adults with acute sinusitis. A systematic review. J GenIntern Med 5/2003 18(5):390–401 10.1046/j.1525-1497.2003.20744.x PMC1494859 12795739

