# Supplementary material for: Patient-centered outcomes for clinical trials in chronic rhinosinusitis with or without nasal polyps and allergic fungal rhinosinusitis
Source: J Patient Rep Outcomes. 2025 Jan 23;9:11. doi: 10.1186/s41687-024-00833-6 (PMC11757837; doi:10.1186/s41687-024-00833-6)
Supplement: Supplementary file 1 — Supplementary Material 1 [file 41687_2024_833_MOESM1_ESM.docx]

Appendix A: Supplementary materials

[1 Concept-focused literature review search strategies 2](#_Toc177999691)

[2 Description of previously identified clinical outcomes assessments (COAs) 5](#_Toc177999692)

[3 Literature search flow diagrams by condition 6](#_Toc177999693)

[4 COA landscape inquiry exclusions 9](#_Toc177999694)

# Concept-focused literature review search strategies

*CRSsNP*

The goal of the concept-focused literature review is to identify, describe, and substantiate concepts of measurement that reflect the primary signs, symptoms, and impacts associated with CRSsNP from the perspective of the empirical literature in adults and adolescents. To accomplish this goal, the following objectives are specified:

- Develop a targeted search strategy, presented in Table 1. Concept-focused literature review search criteria, to identify empirical literature that presents relevant concepts of measurement related to AFRS in adult and adolescent patients;
- Conduct a review of identified abstracts (up to 200 total), as well as a supplementary grey literature search, to identify published literature that presents concepts of measurement related to CRSsNP in adult and adolescent patients; and
- Conduct a full-text review of selected publications (up to 15 total).

This search was conducted on 19 August 2022 in OVID SP, using MEDLINE®, PsycINFO, and Embase.

Table 1. Concept-focused literature review search criteria

| Step | Search terms | Search field | Number of results |
| --- | --- | --- | --- |
|  | Chronic Rhinosinusitis without Nasal Polyp$1 OR CRSsNP | Abstracts | 1,398 |
|  | Symptom* OR sign$1 OR health related quality life OR HRQOL OR HRQL OR QoL OR quality life OR impact* OR emotion* OR social OR physical OR (activit* ADJ3 daily li*) OR work OR school OR qualitative OR patient report* | Abstracts | 12,824,609 |
|  | (1) and (2) | -- | 463 |
|  | Limit (3) to English language | -- | 431 |
|  | Limit (4) to Human | -- | 400 |
|  | Limit (5) to published in the last 10 years (“2012 – Current”) | -- | 359 |
|  | Duplicates removed from (6) | -- | 226 |

Databases included were Embase 1996-2022 Week 32, MEDLINE(R) ALL 1946 to August 18, 2022, and APA PsycINFO 2002 to August Week 3 2022

* indicates unlimited truncation (i.e., performs searches for variations on a word that are formed with different suffixes)

adj indicates the use of the Defined Adjacency Operator, which retrieves documents that contain query words within a specified number (n) of words from each other in any order

$1 indicates limiting truncation to one letter at end of search term

*CRSwNP*

The goal of the concept-focused literature review is to identify, describe, and substantiate concepts of measurement that reflect the primary signs, symptoms, and impacts associated with CRSwNP from the perspective of the empirical literature in adults and adolescents. To accomplish this goal, the following objectives are specified:

- Develop a targeted search strategy, presented in Table 1. Concept-focused literature review search criteria to identify empirical literature that presents relevant concepts of measurement related to CRSwNP in adult and adolescent patients;
- Conduct a review of identified abstracts (up to 200 total), as well as a supplementary grey literature search, to identify published literature that presents concepts of measurement related to CRSwNP in adult and adolescent patients; and
- Conduct a full-text review of selected publications (up to 15 total).

This search was conducted on 19 August 2022 in OVID SP, using MEDLINE®, PsycINFO, and Embase.

Table 1. Concept-focused literature review search criteria

| Step | Search terms | Search field | Number of results |
| --- | --- | --- | --- |
|  | Chronic Rhinosinusitis with Nasal Polyp$1 OR CRSwNP | Keyword | 539 |
|  | Symptom* OR sign$1 OR health related quality life OR HRQOL OR HRQL OR QoL OR quality life OR impact* OR emotion* OR social OR physical OR (activit* ADJ3 daily li*) OR work OR school OR qualitative OR patient report* | All fields | 25,782,370 |
|  | (1) and (2) | -- | 318 |
|  | Limit (3) to English language | -- | 311 |
|  | Limit (4) to Human | -- | 264 |
|  | Limit (5) to published in the last 10 years (“2012 – Current”) | -- | 262 |
|  | Duplicates removed from (6) | -- | 168 |

Databases included were Embase 1996-2022 Week 32, MEDLINE(R) ALL 1946 to August 18, 2022, and APA PsycINFO 2002 to August Week 3 2022

* indicates unlimited truncation (i.e., performs searches for variations on a word that are formed with different suffixes)

adj indicates the use of the Defined Adjacency Operator, which retrieves documents that contain query words within a specified number (n) of words from each other in any order

$1 indicates limiting truncation to one letter at end of search term

*AFRS*

The goal of the concept-focused literature review is to identify, describe, and substantiate concepts of measurement that reflect the primary signs, symptoms, and impacts associated with AFRS from the perspective of the empirical literature in adults and adolescents. To accomplish this goal, the following objectives are specified:

- Develop a targeted search strategy, presented in Table 1, to identify empirical literature that presents relevant concepts of measurement related to AFRS in adult and adolescent patients;
- Conduct a review of identified abstracts (up to 200 total), as well as a supplementary grey literature search, to identify published literature that presents concepts of measurement related to AFRS in adult and adolescent patients; and
- Conduct a full-text review of selected publications (up to 15 total).

This search was conducted on 19 August 2022 in OVID SP, using MEDLINE®, PsycINFO, and Embase.

Table 1. Concept-focused literature review search criteria

| Step | Search terms | Search field | Number of results |
| --- | --- | --- | --- |
|  | Allergic Fungal Rhinosinusitis OR AFRS | All fields | 1,022 |
|  | Symptom* OR sign$1 OR health related quality life OR HRQOL OR HRQL OR QoL OR quality life OR impact* OR emotion* OR social OR physical OR (activit* ADJ3 daily li*) OR work OR school OR qualitative OR patient report* | All fields | 25,782,370 |
|  | (1) and (2) | -- | 528 |
|  | Limit (3) to English language | -- | 516 |
|  | Limit (4) to Human | -- | 397 |
|  | Limit (5) to published in the last 10 years (“2012 – Current”) | -- | 317 |
|  | Duplicates removed from (6) | -- | 209 |

Databases included were Embase 1996-2022 Week 32, MEDLINE(R) ALL 1946 to August 18, 2022, and APA PsycINFO 2002 to August Week 3 2022

* indicates unlimited truncation (i.e., performs searches for variations on a word that are formed with different suffixes)

adj indicates the use of the Defined Adjacency Operator, which retrieves documents that contain query words within a specified number (n) of words from each other in any order

$1 indicates limiting truncation to one letter at end of search term

# Description of previously identified clinical outcomes assessments (COAs)

The SNOT-22 is a 22-item self-reported questionnaire assessing symptoms and social/emotional impacts of the patient’s rhinosinusitis using a recall period of “the past two weeks”, while the ACQ-6 is a six-item self-reported questionnaire which assesses impacts of asthma with a recall period of “the past week”. The UPSIT is a 40-item task administered to assess ability to identify odors, where participants are asked to match odor names to their corresponding scratch-n-sniff labels within a booklet. The development articles for the SNOT-22, ACQ-6, and UPSIT are each cited within the manuscript. The Rhinosinusitis VAS assesses overall severity of rhinosinusitis by asking “How troublesome are your symptoms of your rhinosinusitis” on a 10-cm VAS from 0 (‘not troublesome’) to 10 (‘worst thinkable troublesome’) (developed by Sponsor for use in clinical trials; clinical trial records are cited within manuscript). The Nasal Symptom Diaries for each condition assess the severity of CRS nasal symptoms (nasal congestion/obstruction, loss of smell, anterior rhinorrhea and posterior rhinorrhea) on a daily basis (developed by Sponsor for use in clinical trials; clinical trial records are cited within manuscript).

# Literature search flow diagrams by condition

Figure 1. CRSsNP literature search flow diagram


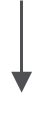

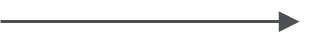

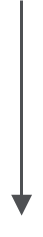

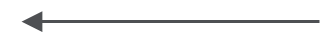

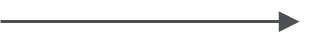


Citations identified in MEDLINE®, Embase, and PsycINFO® search (via OVID SP)

(n=226)

Abstracts selected for

full-text review (n=10)

Total full-text articles included in the final literature analysis (N=12)

Abstracts that did not meet the inclusion criteria (n=216)

Relevant publications identified via supplemental literature search (n=3)

Articles excluded due to lack of relevant data following initial full-text review (n=1)

Figure 2. CRSwNP literature search flow diagram


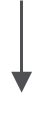

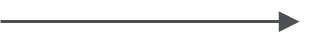

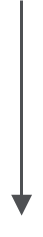

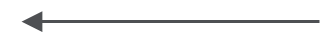

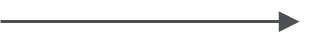


Citations identified in MEDLINE®, Embase, and PsycINFO® search (via OVIDSP)

(n=168)

Publications selected for

full-text review (n=7)

Total publications included in the final literature analysis (N=20)

Abstracts that did not meet the inclusion criteria (n=161)

Relevant publications identified via supplemental literature search and reference review (n=15)

Publications excluded due to lack of relevant data following initial full-text review (n=2)

Figure 3. AFRS literature search flow diagram


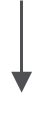

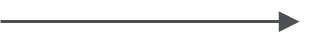

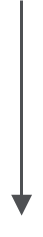

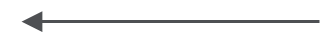

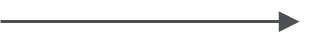


Citations identified in MEDLINE®, Embase, and PsycINFO® search (via OVIDSP)

(n=209)

Publications selected for

full-text review (n=7)

Total publications included in the final literature analysis (N=6)

Abstracts that did not meet the inclusion criteria (n=202)

Relevant publications identified via supplemental literature search (n=6)

Publications excluded due to lack of relevant data following initial full-text review (n=7)

# COA landscape inquiry exclusions

*CRSsNP*

Of the 14 CRSsNP clinical trials identified in the clinical trial search, three were excluded due to focus in non-CRSsNP patient populations, which resulted in the review of 11 trials for use of COAs. The one identified FDA label was excluded due to not being indicated for treatment of CRSsNP.

*CRSwNP*

Of the 17 clinical trials identified in the clinical trial search, three were excluded due to non-CRSwNP patient populations, which resulted in the review of 14 trials for use of COAs. To note, one trial did not describe use of any COAs.
